# Path-Dependent Hydration and Dehydration of CaCl_2_

**DOI:** 10.1021/acs.cgd.4c00628

**Published:** 2025-07-11

**Authors:** Michaela C. Eberbach, Hyerin Seo, Aleksandr I. Shkatulov, Paul Tinnemans, Hendrik P. Huinink, Hartmut R. Fischer, Olaf C. G. Adan

**Affiliations:** † 3169 Eindhoven University of Technology, Den Dolech 2, Eindhoven 5600 MB, The Netherlands; ‡ EIRES, Horsten 1, Eindhoven 5612 AZ, The Netherlands; § Iberian Center for Research in Energy Storage, CIIAE, Polígono 13, Parcela 31, “El Cuartillo”, Cáceres 10004, Spain; ∥ Radboud University, Houtlaan 4, Nijmegen 6525 XZ, The Netherlands; ⊥ TNO Materials Solutions, High Tech Campus 25, Eindhoven, 5656 AE, The Netherlands

## Abstract

In heat storage research, CaCl_2_ is one of the most often used salt hydrates due to its abundance, low costs, and the possibility of a high water vapor uptake. However, the phase transitions among the lower hydrates (0, 1/3, 1, 2) have been discussed since the discovery of CaCl_2_·1/3H_2_O (hereinafter called “tritohydrate”) and the dependency of (de)­hydration steps on the reaction path. This research addresses the reasons behind this path dependency and the position of the tritohydrate in the phase diagram. With TGA experiments, we show that the hydration pathway is 0–1/3–2 and the dehydration pathway is 2–1–0 at low water vapor pressures and 2–1/3–0 at high vapor pressures. It is shown that poor kinetics of the 1/3–1 transition and the metastability of the tritohydrate cause this. We have resolved the crystalline structures of the tritohydrate and monohydrate, which makes clear that a 1/3–1 transition involves large rearrangements of the crystalline structure. Consequently, steps from 1/3–1 hydration and 1–1/3 dehydration will have a high activation energy, hampering these transitions.

## Graphical abstract

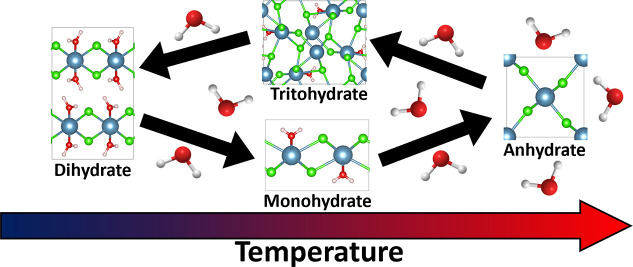

## Introduction

Due to climate change,^1–4^ there is a current shift toward renewable energy sources.^5^ Unlike fossil fuels, which provide a steady energy supply, renewable sources like solar power encounter challenges due to their peak production times not always aligning with peak demand, especially during mornings, evenings, and winters.^6–8^ To address this production–demand mismatch, energy storage devices come into play.^9–11^ These devices are charged during periods of high production and low demand and discharged when the situation is reversed. One type of energy storage is thermal energy storage (TES) or heat batteries, which can find applications in space heating, hot tap water in residential homes, or energy recovery in industrial processes requiring or releasing thermal energy or heat.

There are various types of TES, including sensible, latent, thermochemical, and sorptive.^12,13^ While sensible and latent energy storage techniques are well understood and are already in use, they have a lower potential energy density compared to thermochemical energy storage (TCES) materials.^14^ The sensible TES has an energy storage density of around 0.2 GJ/m^3^,^15,16^ latent TES has a higher storage density of around 0.3–0.4 GJ/m^3^,^15,16^ and TCES has the highest maximal storage density of around 1–3 GJ/m^3^.^15,16^ Additionally, TCES systems can store energy with zero losses, which makes this method the preferred choice for long-term and seasonal storage, essential for balancing renewable energy production differences between summer and winter.

The required temperature regimes for storage materials vary depending on the application. For residential homes, low-temperature systems (<100 °C)^17^ are necessary. However, other applications, such as industrial waste heat storage or upgrading, require higher temperatures.

The storage materials often considered for long-term low-temperature TCES for residential use are salt hydrates. Salt hydrates can be decomposed by waste or renewable heat to release water vapor and turn into anhydrous salts. These anhydrous salts can absorb water vapor to form different hydrate phases and release heat in the process, as described in eq 1, illustrating an example of CaCl_2_ taken as a salt.^17^ Then the cycle was carried out anew. The temperature and energy generated during salt hydration depend on the type of salt used and the water vapor pressure.

1 $${CaCl}_{2}\cdot {aH_{2}O}_{\left(s\right)}+\left(b-a\right)\cdot {H_{2}O}_{\left(g\right)}\Leftrightarrow {CaCl}_{2}{\cdot bH_{2}O}_{\left(s\right)}+\Delta H$$

The equilibrium line of such a (de)­hydration can be described by eq 2 ^18^

2 $$\ln\left(\frac{p_{eq}}{p^{0}}\right)=-\left(\frac{\Delta S_{ab}^{0}}{R}-\frac{\Delta H_{ab}^{0}}{RT}\right)$$

With *p*_eq_ [mbar] representing the equilibrium water vapor pressure, *p* ^0^ [mbar] standing for the standard pressure of 1000 mbar, Δ*S*_ab_^0^ [J/mol K], and Δ*H*_ab_^0^ [J/mol] for the standard molar entropy and the enthalpy of transition CaCl_2_·aH_2_O→CaCl_2_·bH_2_O per mole water and *R* [8.3145 J/mol K] for the ideal gas constant.

There are several phases of CaCl_2_·*x*H_2_O thoroughly characterized in the literature. In 1889, Bakhuis Roozeboom^19^ constructed a phase diagram for the solubility of CaCl_2_ in water, correlating it with the CaCl_2_/H_2_O ratio through solubility measurements as a function of temperature. Together with another tetrahydrate polymorph discovered by Bassett et al.,^20^ seven crystal phases of CaCl_2_·*x*H_2_O were identified: anhydrate (*x* = 0), monohydrate (*x* = 1), dihydrate (*x* = 2), α-tetrahydrate (4α, *x* = 4), β-tetrahydrate (4β, *x* = 4), γ-tetrahydrate (4γ, *x* = 4), and hexahydrate (*x* = 6). In 1936, Lannung^21^ measured equilibrium lines for the hydration (solid–solid) phase transitions as a function of water vapor pressure and temperature of 0–1, 1–2, 2–4α, and 4γ-6 transitions. The choice of transitions involving the tetrahydrate was based on the ease of forming the 4γ polymorph from a saturated solution of the tetrahydrate, even when seeded with hexahydrate crystals. 4α is the most stable polymorph of the tetrahydrate, rapidly formed from 4γ over time. From this stable polymorph 4α, the dihydrate is obtained during heating.

In 1985, the 1/3-hydrate of CaCl_2_ (CaCl_2_·1/3 H_2_O or 3CaCl_2_·H_2_O) was reported by Sinke et al.,^22^ supported by calculations by Pitzer et al. in 1994.^23^ In this work, this hydrate is called tritohydrate due to the Greek “trito for one-third”.^24^ They found that Roozeboom’s solutions’ molality suggests he was measuring the tritohydrate’s solubility instead of the monohydrate. After finding a synthesis method, they established the powder X-ray diffraction (PXRD) pattern of this new tritohydrate phase. Additionally, Pitzer et al. determined the enthalpy of the solution of the tested phases, compared to the work done by Parker et al.^11^ Earlier works on CaCl_2_, SrCl_2_, and BaCl_2_ found fractional hydrates for CaCl_2_ and SrCl_2_ but not for BaCl_2_.^25^ Evidence for fractional hydrates in SrCl_2_ and BaCl_2_ was discovered, assumed to be hemihydrates.^26–28^ However, nothing similar to the tritohydrate of CaCl_2_ was found. This shows that crystalline hydrates with a fractional number of water molecules per formula unit of salt are possible, but they are more commonly hemihydrates than 1/3-hydrates like those found for CaCl_2_.

In 2013, Molenda et al.^29^ observed the tritohydrate during isobaric hydration and dehydration cycles of CaCl_2_ at different water vapor pressures. They showed that during hydration, the only intermediate phase between the anhydrate and dihydrate contained around 0.3 mol of H_2_O per mol of salt, similar to the results from Sinke et al.^22^ The intermediate phase during dehydration from the dihydrate depends on the water vapor pressure. Below 100 mbar, the intermediate phase was the monohydrate, while above 100 mbar was the tritohydrate. Around 100 mbar of water vapor pressure, a mixture of phases of monohydrate and tritohydrate was observed.

The papers about CaCl_2_ hydrate phase diagrams^19,21–23,29^ indicate hydration transitions of the tritohydrate of CaCl_2_ are only partially known. Various studies, such as those by Bakhuis-Roozeboom and Sinke et al., have determined the solubilities of different hydrates, offering some insights into their positions in a phase diagram. However, solid–solid phase transitions differ from solid–liquid transitions and can occur over a wide range of temperatures and water vapor pressures, often far from their liquid counterparts. Lannung determined some equilibrium lines for solid–solid phase transitions, but this was before tritohydrate was discovered. Consequently, the equilibrium lines for the 0–1/3 and 1/3–2 transitions remained unknown.

In addition to the equilibrium lines, salt hydrates can have hydration and dehydration onsets during kinetic measurements called metastable zone (MSZ) boundaries, as described by Sögütoglu et al.^30^ These onsets surround a region close to the equilibrium line, with slow kinetics and induction times. These MSZs lower the temperature that a salt hydrate can generate during hydration and/or increase the temperature required to dehydrate it again. Not all transitions of salt hydrates have MSZs, and some MSZs are pronounced only on the hydration side or only on the dehydration side (e.g., LiCl–LiCl·H_2_O transition^30^) of the equilibrium line, while others can be wide on both sides (e.g., transitions of CuCl_2_ and K_2_CO_3_ to their respective hydrates^30^). Molenda et al. conducted kinetic studies on CaCl_2_, as noted in ref 29 but did not establish complete hydration and dehydration onset lines or MSZs. Furthermore, the crystal structures of both the trito- and monohydrate are unknown, in contrast to the anhydrate,^31,32^ dihydrate,^33^ hexahydrate,^34^ and polymorphs of the tetrahydrate.^35–37^

The goal of this paper is to understand the hydration–dehydration pathways of the lower CaCl_2_ hydrates (0, 1/3, 1, 2) at low, medium, and high water vapor pressures via the comprehensive thermal and structural characterization of CaCl_2_ and its hydrates. Therefore, TGA measurements at various partial vapor pressures and heating/cooling rates were performed for 0–2 cycles with multiple transitions and single transition cycles (0–1/3 and 1–2 cycles) to determine the conditions for the onsets. To confirm the phases during these kinetic cycles, powder X-ray diffraction (PXRD) in situ measurements were used. This work reports for the first time the crystal structures of the mono- and tritohydrate, which were determined by using single-crystal X-ray diffraction (SCXRD) on single crystals grown in the respective phase in autoclaves.

## Materials and Experimental Methods

### Materials

Calcium dichloride was supplied by Sigma-Aldrich in the form of the dihydrate (CaCl_2_·2H_2_O, ACS reagent, ≤99.0%) and from VWR International B.V. in the form of the hexahydrate (CaCl_2_·6H_2_O, ThermoFisher Scientific, 98 + % for analysis). For use as a TGA sample, CaCl_2_·2H_2_O was preliminarily ground very lightly in a mortar that was preheated in a 160 °C oven to avoid the deliquescence of the salt.

Single crystals of the tritohydrate (CaCl_2_·1/3H_2_O) and monohydrate (CaCl_2_·H_2_O) were synthesized in an in-house-built autoclave by using the phase diagram given by Sinke et al.^22^ for guidance (Figure 1a). Therefore, 1.5 to 1.9 mol of H_2_O per mol of CaCl_2_ (=1.217–1.518 mL) was mixed with 5 g of CaCl_2_ anhydrate inside a 100 mL Teflon bottle. The Teflon bottle was put inside an in-house-built autoclave as shown in Figure 1b, which was then closed airtight. The autoclave was first heated to 250 °C for 24 h before it was cooled to either 210 °C for the tritohydrate or 180 °C for the monohydrate. This temperature was then maintained for 5 days. Afterward, the autoclave was cooled down as fast as possible by exposing it to the ambient atmosphere, which took a few hours (∼2– 6 h) due to the large mass of the device. The cooled autoclave was opened again, and the Teflon bottle was sealed with parafilm for transport to the Single Crystal XRD (SCXRD) at Radboud University Nijmegen. The SCXRD was then used to determine the crystal structure of these two hydrates.

**Figure 1:**
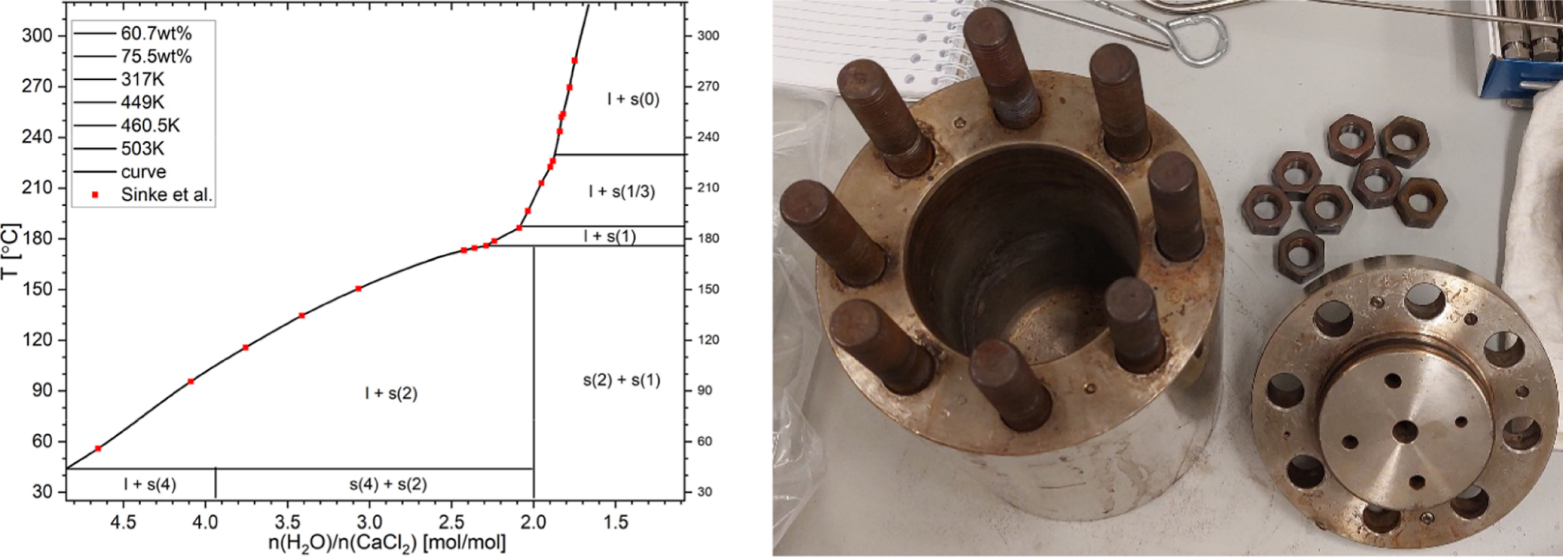
(a) The solubility phase diagram of the different CaCl_2_ hydrate phases adapted from ref 22 as a function of the temperature and the composition between the water molecules and salt ion pairs inside the sample, which Bakhuis Roozeboom constructed^19^ and improved and Sinke et al. refined in 1985.^22^ (b) The in-house-built autoclaves used to synthesize the single crystals of CaCl_2_ trito- and monohydrate.

### Isobaric TGA Cycles

Isobaric water sorption and desorption of the pure CaCl_2_ salt were investigated using thermogravimetric analysis on two TGA devices, Mettler Toledo TGA/SDTA851e and Mettler Toledo TGA/DSC 3+, as described in ref 38. The two TGA setups were used with a home-built and a Cellkraft humidifier, respectively. The oven temperature could be controlled between 25 and 1000 °C and recorded together with the sample temperature. The sample was located in both devices on a balance arm inside an oven, with a weight measurement accuracy of ±1 μg.

Both machines were operated under ambient pressure and had an inlet for gas flow connected to one of the humidifiers. The home-built humidifier was operated at 18 °C, and a dry (0% RH) and a wet (100% RH) N_2_ flow were mixed to generate a water vapor pressure between 0 and 20 mbar. This was done by Arduino-supported mass flow controllers, which mixed the two flows in different ratios to create the desired water vapor pressure. This home-built device was connected to the TGA/SDTA851e. The second humidifier was a Cellkraft Humidifier P-2 operating at 25 °C, which worked via a feedback loop from an RH sensor at the outlet of the humidifier and was connected to the TGA/DSC 3+. Both devices had a 300 mL/min flow rate over the sample inside the TGAs.

The temperatures of both TGAs were calibrated to an accuracy of 0.2 K using the melting points of naphthalene, indium, and zinc.^39^ The humidifiers were calibrated to an accuracy of ±0.16 mbar water vapor pressure in the N_2_ flow using the gravimetric signal at the deliquescence point of LiCl·H_2_O, CH_3_COOK, K_2_CO_3_·1.5 H_2_O, MgCl_2_·6H_2_O, and Mg­(NO_3_)_2_·6H_2_O at 25 °C^18,40^ due to the uncertainty in determining the deliquescence onset by the weight change corresponding to the humidity step.

For measurements at high water vapor pressures, the NETZSCH 449 F3 Jupiter TGA was equipped with a vapor generator. The water vapor furnace was used along with the TGA sample holder with a K-type thermocouple (up to 700 °C). The accuracy of the weight measurement was ±0.1 μg. The humidity was generated with the proUmid MHG100 humidifier at the saturation temperature of 82.5 °C to generate 50, 100, 200, 400, and 700 mbar of partial water vapor pressure in the N_2_ flow.

A temperature program was run with a sample while the humidifier was turned on simultaneously at the desired water vapor pressure. Thereby, a program usually consisted of an isothermal step at a high temperature to ensure complete dehydration, followed by cooling at a certain K/min rate, an isothermal step at the lowest temperature to ensure full hydration, and then heating at the same rate as that with cooling back to the high temperature, which was again held in an isothermal step to ensure full dehydration again. The resulting weight changes were used to calculate a parameter called loading *L* [mol H_2_O/mol CaCl_2_], which is the amount of water in moles per mol salt. The loading was calculated similarly to ref 18 using the dry weight at high temperatures of each sample and the current weight

3 $$L=\frac{m-m_{a}}{M_{w}}\cdot \frac{M_{CaCl2}}{m_{a}}$$

Here, *m* [g] stands for the current mass of the sample, *m*_a_ [g] for the sample mass of the anhydrate, *M*_w_ [g/mol] for the molecular mass of water (18.02 g/mol), and *M*_CaCl2_ [g/mol] for the molecular mass of calcium­(II) dichloride anhydrate (111 g/mol).

Additional measurements were performed to confirm the formation of the monohydrate during hydration. First, three precycles were done between 50 and 125 °C with a heating/cooling rate of 1 °C/min and water vapor pressure maintained at 12 mbar. The actual measurement was done in a fourth cycle that was performed with a 0.1 °C/min cooling rate. While cooling the sample in this fourth cycle, the experiment was paused five times during 12 h at various temperatures *T* = 67, 65, 63, 61, and 59 °C. The pauses were done to observe in which direction the hydration process would proceed under isothermal conditions. Finally, the sample was heated again at a rate of 0.1 °C/min back to 125 °C.

#### Powder X-ray Diffraction (PXRD) Analysis In Situ

As described in ref 38, PXRD was performed using a Rigaku Mini-Flex diffractometer in continuous scan mode with a divergent slit of 0.625° and a D/teX Ultra2 detector, using Cu K_α_-radiation and K_β_ filter. To identify the crystalline phases of the different salt hydrates and to observe the phase transitions, in situ measurements were performed using an Anton Paar BTS 500 heating stage, which is built into the diffractometer, and an attached humidifier, which can blow nitrogen (800 mL/min) with 0–20 mbar water vapor over the sample. The measurement was carried out with Bragg–Brentano geometry at 2θ = 5–75° with step sizes between 0.005° and 0.01° and a speed of 1° to 10°/min. The humidifier worked similarly to the home-built humidifier of the TGAs, but the flow rate was set to 800 mL/min because of the larger sample size.

Two types of PXRD measurements were performed. First, a detailed scan of each CaCl_2_ hydrate was performed. This was done under the conditions mimicking the preparation conditions of the respective hydrate samples, for example, 120 °C and 0 mbar for the anhydrate and no temperature control, i.e., room temperature, and 6 mbar for the tetrahydrate or 9 mbar for the hexahydrate. The measurement settings were with the 2θ ranging from 10° to 75°, a step size of 0.005°, and a scanning rate of 3°/min. Each of these scans took around 25 min next with the temperature being held constant to ensure complete dehydration for the anhydrate sample.

Second, isobaric in situ measurements were performed using several scans at different temperatures with a constant partial water vapor pressure *p*_vap_ = 12 mbar. Since the in situ measurements needed several scans (30–50 scans depending on the set program), the scans were shortened to reduce the measurement time. Considering that the strongest reflection of the anhydrate and different hydrates lies between 10° and 50°, the range of 2θ was shortened from 10–75° to 10–50° with a step size of 0.050° and a scanning rate of 5°/min, which changed the recording time of one scan to 9 min.

The isobaric in situ PXRD measurement was executed as follows. First, the sample was brought to a starting temperature of 150 °C when the first scan was recorded, and the sample was cooled down to 104 °C with a 1 K/min cooling rate. Then, the temperature decreased in steps of 2 K starting from 104 °C and ending at 60 °C. A diffractogram was recorded at each temperature step. Afterward, the sample was cooled to 45 °C at 1 K/min. The diffractogram at the lowest temperature of 45 °C was measured with a subsequent increase in temperature from 84 to 130 °C in steps of 2 K and an end temperature of 150 °C. The start, lowest, and end temperatures were held for 3 h before the reflections were recorded to ensure that the sample has completely transitioned, while all other temperatures were only held for either 1 or 30 min before the recording of the reflections, for an experiment similar to the TGA measurements or slow measurements to get clearer patterns of the intermediate phases.

Additional in situ measurements were performed to mimic the TGA measurements to confirm the monohydrate formation during hydration. Therefore, the sample was precycled between 45 and 150 °C at 1 K/min for three cycles at P­(H_2_O) = 12 mbar. Then in the fourth cycle, the sample was cooled from 150 to 80 °C with a rate of 1 K/min. At the start temperature (150 °C) and end temperature (80 °C), a diffractogram was measured. The sample was kept at 80 °C for 12 h, and later, a diffractogram was measured. Afterward, the sample was cooled to 65 °C, and another diffractogram was recorded. This temperature was held for 12 h, and the next diffractogram was recorded. At the end, the sample was cooled to 45 °C. The sample was scanned again, and the temperature was held for 3 h. Finally, the last scan of this measurement was performed.

#### Single-Crystal X-ray Diffraction (SCXRD)

The crystal structures of the CaCl_2_ tritohydrate and monohydrate were determined by performing SCXRD on the crystals grown in an autoclave, as described above. Before the measurements, the crystals were coated with oil to prevent the hydration of the salt. Reflections were observed on a Bruker D8 Quest diffractometer with a sealed tube and Triumph monochromator (λ = 0.71073 Å). The software package used for the intensity integration was Saint (v8.40a).^41^ Absorption correction was performed with SADABS.^42^ The structures were solved with direct methods using SHELXT-2014/5.^43^ Least-squares refinement was performed with SHELXL-2018/3 ^44^ against all reflections. Non-hydrogen atoms were refined freely with anisotropic displacement parameters. Hydrogen atoms were placed on calculated positions or located in difference Fourier maps. All calculated hydrogen atoms were refined with a riding model.

## Results and Discussion

### (De)­Hydration Pathways at 12 mbar: TGA

To comprehend the phase transitions between CaCl_2_ hydrates during cooling (hydration) and heating (dehydration), it is crucial to determine the onset temperatures of these transitions. So, as a first test, an isobaric TGA measurement at a 12 mbar water vapor pressure from 140 to 45 °C and back was performed at a slow temperature ramp of ±0.1 K/min and with a waiting time of 2 h at the highest (140 °C) and lowest temperature (45 °C). The results of this measurement are shown in Figure 2a, with the loading calculated from the weight and weight change of the sample as shown in eq 3. An anhydrous CaCl_2_ is cooled starting from 140 °C at a 12 mbar water vapor pressure and remains anhydrous until 91 °C when it takes up around 0.3 mol of H_2_O/mol of CaCl_2_. The loading remains constant until the temperature reaches 70 °C when the salt absorbs more water vapor until a total of 2 mol of H_2_O/mol CaCl_2_ is reached. This loading remains constant during the rest of the cooling process to 45 °C. Upon heating, the loading remains constant until 81 °C when 1 mol of H_2_O/mol CaCl_2_ is released again, thus resulting in monohydrate CaCl_2_·H_2_O. The remaining water molecules are released when the heating reaches 110 °C. Afterward, the salt persists as an anhydrate for the remaining heating back up to 140 °C. The difference in pathways between hydration and dehydration raises the following questions.

I. Are the observed intermediate phases pure hydrates or mixtures of several hydrate phases?
II. Do the hydration–dehydration steps depend on partial water vapor pressure?
III. What are the single-crystal structures of the intermediate phases, and how does their knowledge help us to understand the path dependency of the steps?
IV. What are the reasons behind the different pathways?

**Figure 2:**
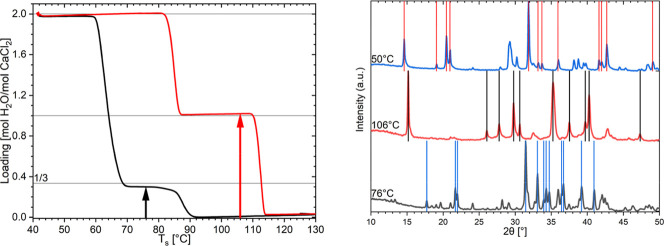
(a) The sample loading during hydration (black) and dehydration (red) of CaCl_2_. The black and red arrows indicate the position where the in situ PXRD diffractograms of the intermediates were taken. (b) The in situ PXRD diffractograms of the CaCl_2_ intermediates at 12 mbar. The diffractograms during hydration at 76 °C are given as the black curve, the one during dehydration at 106 °C in red, and the one in between at the lowest temperature of 50 °C in blue. The literature values of reflections with at least 10% of the maximum intensity for the tritohydrate^22^ (blue), the monohydrate^45^ (black), and dihydrate^33^ (red) are given as vertical lines.

### The Crystalline Phases during (De)­Hydration: PXRD

To address question number (I), PXRD in situ measurements were performed similarly to the TGA cycle. The reflections for the anhydrate and dihydrate can be derived from their crystal structures.^31–33^ For the tri- and monohydrate, however, no crystal structure could be found in the COD, CCDC, ICDD PDF-4+, and AMCSD structural databases. Still, powder diffractograms were reported by Sinke et al.^22^ and Hanawalt et al.,^45^ respectively. These diffractograms and the predicted reflections from the crystal structures could then be used to identify the different phases in the PXRD in situ measurement. Therefore, the finely ground salt was first heated at a 12 mbar water vapor pressure to a temperature of 150 °C and held there for 3 h to ensure the whole sample was completely dehydrated. In the same way as during the TGA measurement (Figure 2a), the phase present in the sample at this high temperature was the anhydrate phase.^31,32^ Then, the sample was cooled at a rate of 1 K/min down to 50 °C. This cooling rate was chosen instead of 0.1 K/min, like in the TGA measurement due to the limitations of the heating stage in the PXRD device, which cannot handle slower temperature changes. The faster temperature ramp was compensated for by the waiting time and the scan time at each temperature step. From 104 to 60 °C, the temperature ramp was interrupted every 2 K to measure a diffractogram of the current crystal phase of the CaCl_2_. During this cooling, the sample hydrated to a phase that coincides well with the PXRD diffractogram of the tritohydrate,^22^ which is shown as the black curve in Figure 2b for 76 °C (black arrow in Figure 2a). At 50 °C, the temperature was held constant again to ensure complete hydration of the whole sample. Here at the 50 °C plateau, the sample fully converted to the dihydrate phase (blue curve in Figure 2b).^33^ Afterward, the salt was heated back to 150 °C in the same way as it was cooled with 1 K/min, and 2 K steps were made between 84 and 130 °C. During this heating, the salt dehydrated to a phase that overlapped mostly with the powder XRD diffractogram of the monohydrate.^45^ The diffractogram of this phase at 106 °C (red arrow in Figure 2a) can be seen in Figure 2b as the red curve.

Thus, all of the phases identified by PXRD are identical to the phases inferred from the loadings from the TGA observations under similar conditions. The PXRD diagrams show no overlapping reflections, which confirms that there are only single crystalline phases for each of the plateaus in loading and no mixture of phases for either the tritohydrate during hydration or the monohydrate during dehydration.

### The Influence of the Vapor Pressure on the (De)­Hydration Pathways: TGA

Regarding question (II), whether the phase transition steps are the same under different conditions, the same cycle of hydration and dehydration from anhydrate to dihydrate and back was performed at various water vapor pressures between 2 and 20 mbar and 50, 100, 200, and 400 mbar at 0.2 K/min like Molenda et al.^29^ Results for the cycle at 400 mbar are listed in Figure 3. The TGA results for the other vapor pressures are given in the Supporting Information (Figure S1). Our results confirm the findings of Molenda et al.^29^ At vapor pressures until 100 mbar, a similar pathway is observed as shown in Figure 2a: hydration follows the pathway 0–1/3–2, while dehydration follows the pathway 2–1–0. Above 100 mbar, the observed intermediate phases of the dehydration pathway follow the one shown in Figure 3.

**Figure 3:**
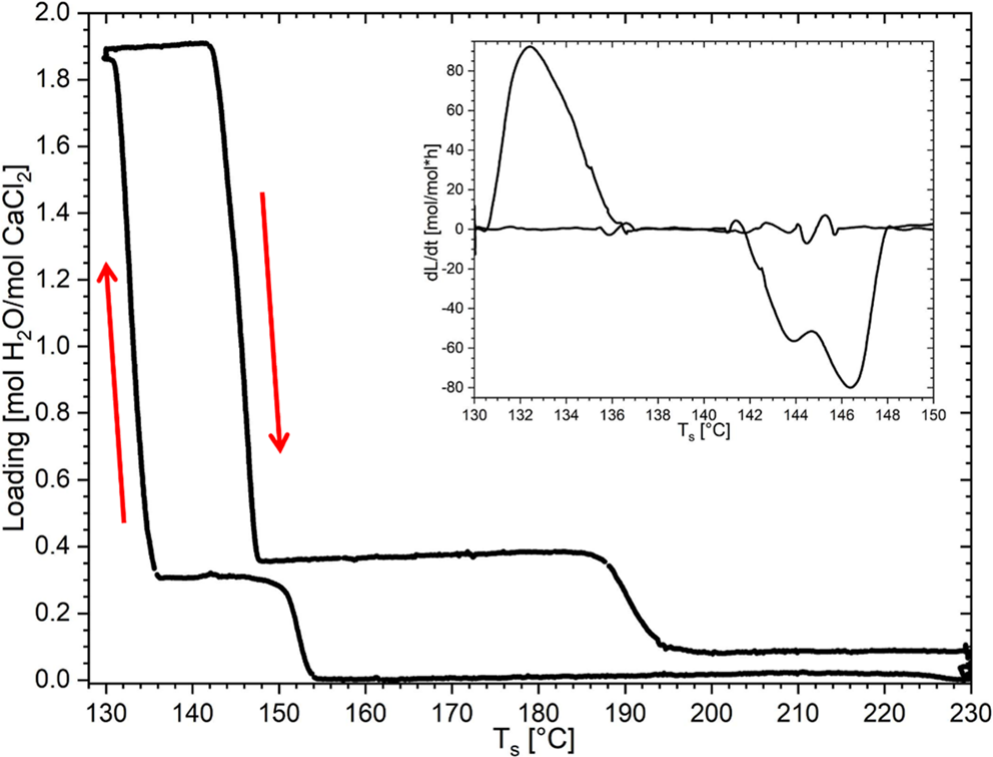
The results of isobaric TGA measurements at 400 mbar, with a heating and cooling rate of 0.2 K/min. The results are represented as the loading of mol of H_2_O per mol of CaCl_2_, and in the smaller insert, the derivative of the loading (L) by the time (*t*) in hours, both as a function of the sample temperature *T*_s_.

An overview of the observed pathways is given in Table 1. The hydration transitions at 400 mbar follow the transitions observed at a low water vapor pressure. During dehydration at high vapor pressures, the steps differ from dehydration at low vapor pressures. Instead of the 2–1–0 pathway, a 2–1/3–0 pathway is observed. When the first derivative of the loading [mol H_2_O/mol CaCl_2_] to time [*h*] is taken of the 400 mbar cycle, it was discovered that the first dehydration step (2–1/3) consists of two peaks (smaller graph in Figure 3). This double peak indicates a two-step process. A two-step process during the 2–1/3 dehydration can be explained by the formation of the monohydrate via the 2–1 dehydration, followed by a 1–1/3 transition, which was earlier reported by Pitzer et al.^23^ So, dehydration above 100 mbar starts in the same way as that at lower water vapor pressures with the 2–1 dehydration (Figure 2a). However, the monohydrate seems to be unstable at these high water vapor pressures or high temperatures. This instability of the monohydrate causes it to transition to the tritohydrate upon formation, which is then visible as the plateau in the TGA measurement. If the same derivative is taken of other phase transitions such as 0–1/3, 1/3–2, 2–1, 1–0, and 1/3–0, then only one peak is obtained with no indications of a double peak.

**Table 1:** The Water Vapor Pressure Regimes for the Different Hydration and Dehydration Paths Are Determined by the Measured Water Vapor Pressures 2–20, 50, 100, 200, and 400 mbar

	hydration	dehydration
*p* = 2–50 mbar	0–1/3–2	2–1–0
p ≈ 100 mbar	0–1/3–2	2–1–1/3 −0
*p* = 200–400 mbar	0–1/3–2	2–1/3 −0

The measurements depicted in Figures 2a and 3 were repeated at multiple water vapor pressures between 2 and 20 mbar and at 50, 100, and 200 mbar. The onsets of all of the observed phase transitions were determined. These onsets are plotted in Figure 4. For comparison to the data points from this work, the equilibrium lines for the 0–1 and 1–2 transitions from Lannung^21^ and the 1/3–1 transition from Pitzer and Oakes^23^ were also plotted in the phase diagram. While Lannung gives a description of his measurements, there is no information on the data points used in Pitzer et al.’s paper, which were taken from unpublished data from G.C. Sinke. Therefore, it is difficult to discuss the reliability of the Pitzer et al. data.

**Figure 4:**
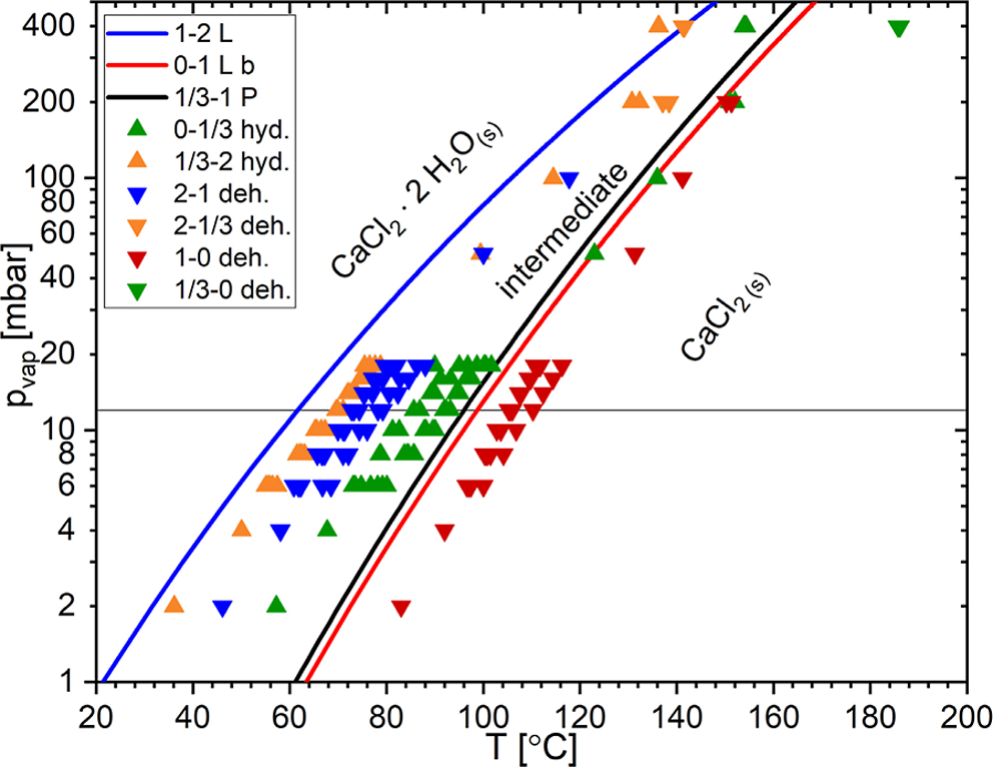
The temperature and water vapor pressure conditions of the phase transition onset from isobaric TGA measurements together with the equilibrium lines from Lannung (“L”)^21^ and Pitzer et al. (“P”).^23^

It is important to notice that the 1/3–2 transition happens before the 1–2 equilibrium line. In this case, the monohydrate would have been a temporary intermediate phase, and one would expect to observe the dihydrate phase at lower temperatures. Before discussing this issue, we first analyze the crystal structures of the trito- and monohydrate in more detail in the next section.

### Single-Crystal Structures: SCXRD

To answer the third question (III), about the single-crystal structures of the intermediates, the COD, CCDC, ICDD PDF4+, and AMCSD databases were searched for information on the unit cells of CaCl_2_ hydrates. Structures for the anhydrate^31,32^ (Figure 5a), dihydrate^33^ (Figure 5d), the three polymorphs of the tetrahydrate,^35–37^ and the hexahydrate^34^ could be found. However, there were no data on the crystal structure of either the tritohydrate or the monohydrate even though they have been known for several decades.

**Figure 5:**
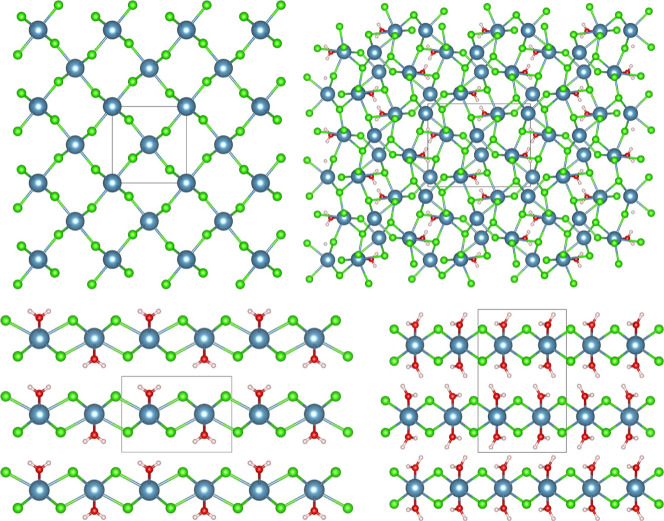
The crystal structures of the unit cells of (a) the anhydrate ab-plane,^31^ (b) the tritohydrate ac-plane (deposition number 2305419), (c) the monohydrate ac-plane (deposition Number 2354016), and (d) the dihydrate bc-plane.^33^

Hence, single crystals of the tritohydrate and monohydrate were grown in an autoclave and analyzed with SCXRD. All details of the crystalline structures can be found in the section Single Crystal Data of Supporting Information (Tables 1 and 2). The crystal structures of the anhydrate and dihydrate from the databases and of the tritohydrate and monohydrate from the SCXRD measurements are shown in Figure 5.

The anhydrate has *Pnnm* symmetry and forms a three-dimensional grid of Ca^2+^ ions surrounded by six Cl^–^ ions that connect them as shown in Figure 5a. Contrary to the anhydrate, the dihydrate with its *Pbcn* symmetry forms two-dimensional planes of Ca^2+^ ions surrounded by four Cl^–^ ions that connect them. The two water molecules are not shared; see Figure 5.

The newly discovered unit cell of the tritohydrate is shown in Figure 5b. It has *Pnma* symmetry. Thereby, it forms a three-dimensional structure of Ca^2+^ ions surrounded by six Cl^–^ ions that connect them, like the anhydrate, and channel-like structures of H_2_O molecules exist along the b-axis, which are connected to some of the Ca^2+^ ions. The H_2_O molecules are each bound to two Ca^2+^ ions and bind to two of three metal ions. The coordination of the Cl^–^ ions around the Ca^2+^ ions in the tritohydrate is not in a polygonal shape as is the case with the ligands in the anhydrate and dihydrate, which both have their ligands (Cl^–^ ions and H_2_O molecules) in an octahedral shape. In the tritohydrate, the shape of the coordinated ligands around a Ca^2+^ ion mostly resembles a “capped trigonal prismatic molecular geometry’’. The unit cell of the tritohydrate has a very large volume: 0.943 nm^3^.

The newly discovered structure of the monohydrate has *Pmmn* symmetry, Figure 5c. Like the dihydrate, it forms two-dimensional sheets of Ca^2+^ ions. However, in the monohydrate, the Ca^2+^ ions are surrounded by six instead of four Cl^–^ and two shared H_2_O molecules. Like in the tritohydrate, the ligand coordination is not ordered in an octahedral shape but mostly resembles a “square antiprismatic’’ because of the eight ligands connected to each metal center.

Contrary to the tritohydrate, which had the largest unit cell of the tested hydrates, the monohydrate unit cell had only a volume of 0.185 nm^3^. This makes the monohydrate unit cell slightly larger than the anhydrate and much smaller than the dihydrate and especially the tritohydrate. Now that the crystal structures of all the examined hydrates are known, the questions about the path-dependent (de)­hydration step of CaCl_2_ can be discussed further.

### The Pathway during Hydration: TGA and PXRD

The fourth question (IV) raised was about the causes for the observed hydration and dehydration pathways, see Figure 2a. In this section, this question is answered for hydration. In the subsequent section, we will address this question for dehydration.

From our hydration experiments, two subquestions appear: (a) why is the monohydrate not observed in the TGA experiments (Figures 2 and 3) and (b) why do we observe the dihydrate phase at temperatures higher than expected based on data of the 1–2 equilibrium line (Figure 4)?

First, we address question (b): why is the dihydrate observed at temperatures higher than expected based on the 1–2 equilibrium? Additional TGA experiments were performed at different water vapor pressures, solely targeting the 1–2 and 0–1/3 transitions. The results for P­(H_2_O) = 12 mbar are shown in Figure 6.

**Figure 6:**
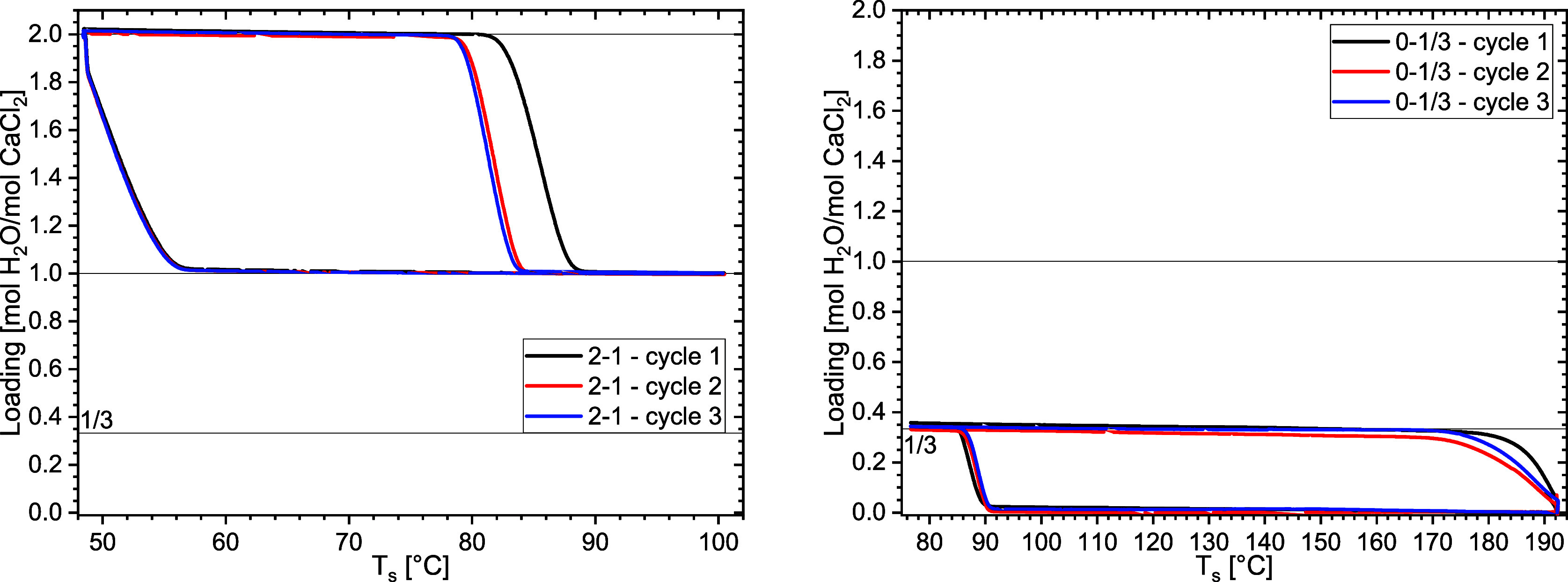
Isobaric TGA measurements at 12 mbar water vapor pressure of (a) the 2–1–2 cycles and (b) the 0–1/3–0 cycles.

The onsets for all 1–2 and 0–1/3 transitions are shown in Figure 7 together with the known phase lines and the previously discussed onsets (see Figure 4).

**Figure 7:**
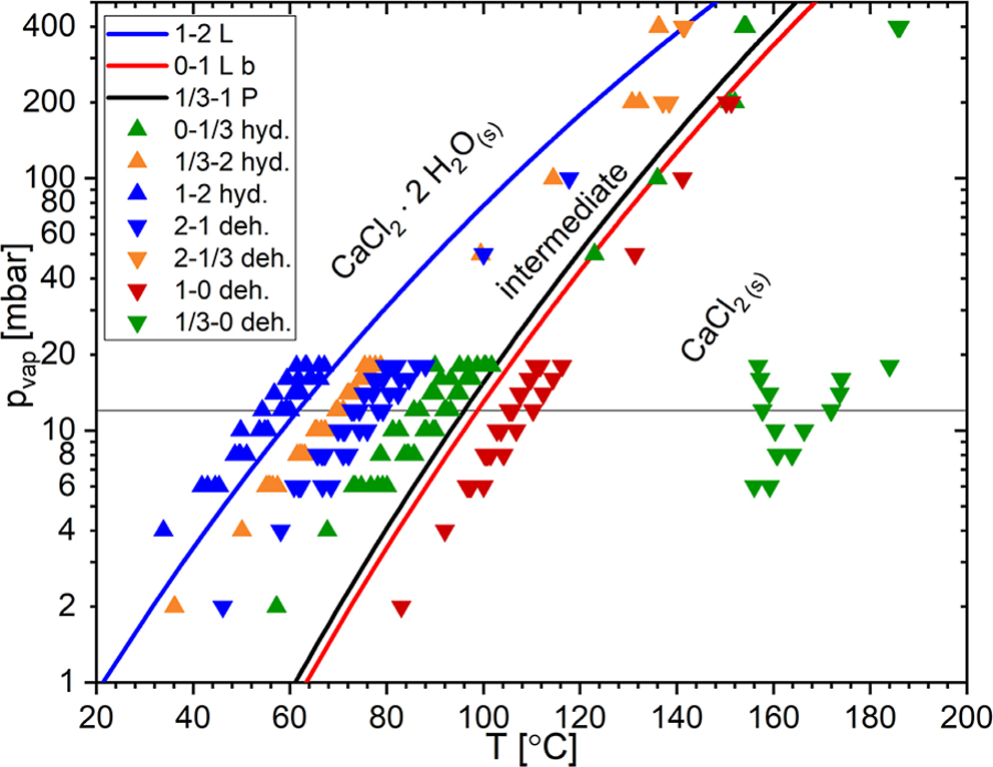
The phase diagram with the temperature–water vapor pressure conditions of the equilibrium lines from Lannung (“L”)^21^ and Pitzer et al.(“P”)^23^ together with onset points for different phase transitions determined in this work.

The observed onset temperatures for the 1–2 hydration transition are located at lower temperatures than the reported 1–2 equilibrium line^21^ due to metastability. Interestingly, the observed 1–2 hydration transition occurs at lower temperatures than the observed 1/3–2 transition. Therefore, it is without doubt that the 1/3–2 hydration step occurs at higher temperatures than the 1–2 step and even the 1–2 equilibrium line. This is peculiar as the solubility phase diagram of the CaCl_2_ determined by Sinke et al.^22^ seems to indicate that there is a stable monohydrate phase. By analyzing the Gibbs free energies of the transitions, one can indeed show that it is possible that a dihydrate can be formed directly from the tritohydrate in a region where the monohydrate is the thermodynamically favored phase. As a starting point, we take the Gibbs free energy change of a transition from a hydrate “*a*” toward a hydrate “*b*” (eq 4), where the numbers *a* and b refer to the number of water molecules per unit salt (eq 1).

4 $$\Delta G_{a\to b}=-RT\left(b-a\right)\ln\left(\frac{p}{p_{eq}^{a\to b}}\right)$$

Here $\Delta G_{a\to b}$ [J/mol] $p_{eq}^{a\to b}$ [mbar] are the Gibbs free energy per mole of salt and the equilibrium water vapor pressure of the hydration *a*→*b*. With this equation, it becomes possible to express the equilibrium vapor pressure of the 1/3–2 transition in terms of the known vapor pressures of the 1/3–1 and 1–2 transitions. As G is a state function, $\Delta G_{1/3\to 2}=\Delta G_{1/3\to 1}+\Delta G_{1\to 2}$ . With the help of eq 4, one can show that

5 $$p_{eq}^{1/3\to 2}={\left(p_{eq}^{1/3\to 1}\right)}^{2/5}{\left(p_{eq}^{1\to 2}\right)}^{3/5}$$

which implies that $p_{eq}^{1/3\to 1}\leq p_{eq}^{1/3\to 2}\leq p_{eq}^{1\to 2}$ . This is precisely what is observed. If the monohydrate is kinetically not accessible, a step from the tritohydrate toward the dihydrate can occur at temperatures and vapor pressures where the monohydrate is thermodynamically the most favorable state. That the dihydrate does not transfer to the monohydrate is a consequence of the broad metastable zone of the 2–1 dehydration reaction.

Therefore, we can also answer the question: why was the monohydrate phase not observed in our TGA measurements (Figure 2)? The 1/3–1 transition is so slow that even with a low cooling rate of 0.1 K/min, it does not have time to develop. This would imply that the development of the monohydrate phase is a matter of time. To test this, we have cooled CaCl_2_ in our TGA and our PXRD to temperatures where we expect the monohydrate to be stable.

TGA measurements were conducted at a water vapor pressure of 12 mbar to investigate the stable hydrate phase between the previously reported 1/3–2 and 1–2 hydration onsets, which is displayed in Figure 8. During long 12 h holds at 67, 65, 63, and 61 °C, the loading stabilized at approximately 1 mol/mol, indicating the formation of the monohydrate phase. This was confirmed through in situ PXRD measurements, where monohydrate phase reflections were observed as shown in the Supporting Information. At 59 °C, an increase in loading was observed, corresponding to the onset of hydration from the monohydrate to the dihydrate. The immediate dehydration upon heating supports this starting hydration transition. This indicates that the monohydrate is the thermodynamically stable phase between 67 and 61 °C but that its formation from the tritohydrate phase is a very slow process. On the contrary, the 1/3–2 transition seems to occur rather fast and therefore outcompetes the 1/3–1 transition in many experiments.

**Figure 8:**
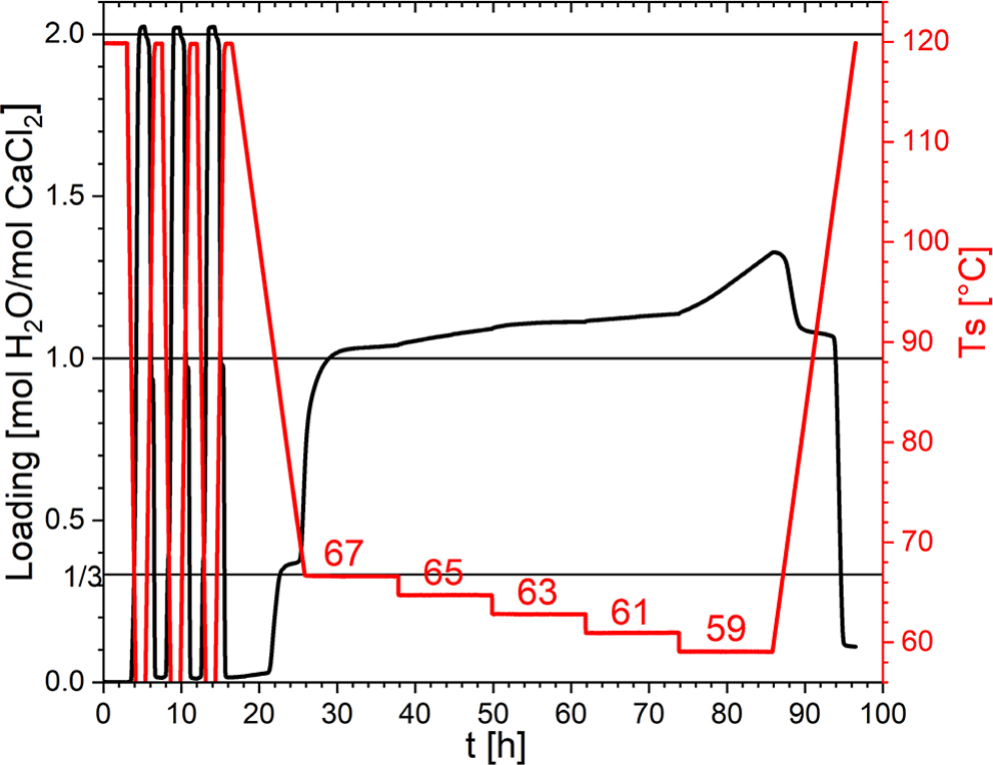
Isobaric TGA measurements at 12 mbar water vapor pressure with three precycles from anhydrate to dihydrate at 1 °C/min and a slow cycle at 0.1 °C/min with 12 h isothermal steps between the 1/3–2 and 1–2 transition onsets.

This picture is further supplemented by DFT calculations of the equilibrium between all four hydrates. In accordance with the equilibrium Δ*G*_*r*_ values calculated at water vapor pressure at 12 and 400 mbar, the equilibrium sequence of phases is 2–1–0 for dehydration and 0–1–2 for hydration (Figure S4). In other words, the DFT calculations predict that the tritohydrate is thermodynamically metastable at both 12 and 400 mbar.

The observation of tritohydrate in hydration and some dehydration experiments, despite thermodynamic metastability, can be explained through the kinetic hindrance of the 0–1 and 1/3–1 transitions. A schematic explanation of the preference for a 1/3–2 transition is given in Figure 9, where the free energy landscape is sketched. Here, the lower activation energy of the 1/3–2 hydration (route: 3–2–4) compared to the higher activation energy of the 1/3–1 hydration (route: 3–1–5) is shown. In this way, the kinetically favorable 1/3–2 hydration transition is easier to perform than the thermodynamically more favorable 1/3–1 hydration transition. In this way, monohydrates are not observed during hydration.

**Figure 9:**
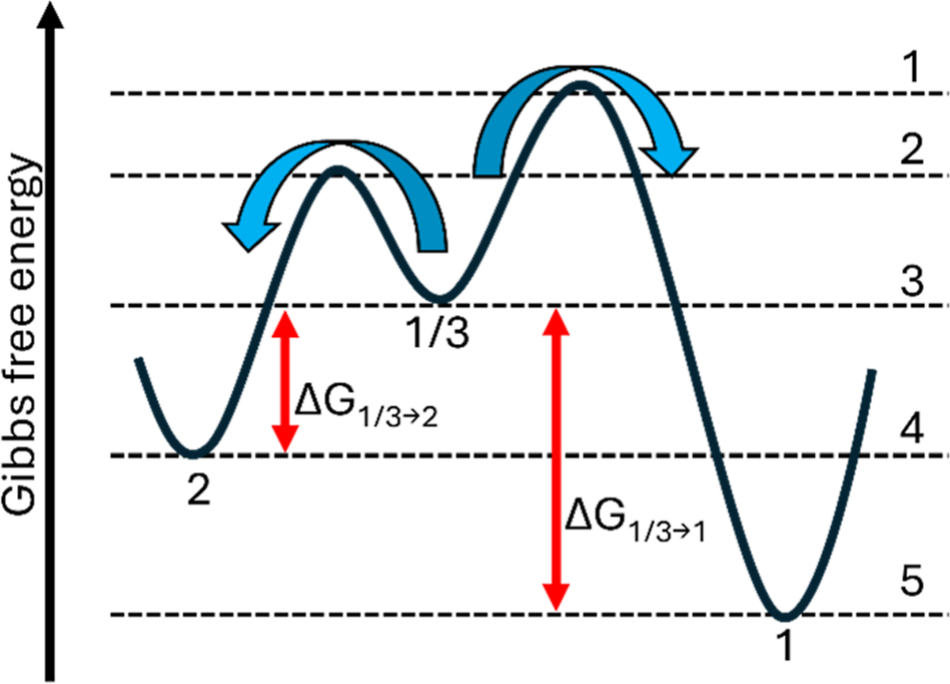
A schematic representation of the Gibbs free energy profile showing the kinetically favorable phase transition of 1/3–2 during hydration.

Finally, to answer question (IV), about the pathway of CaCl_2_ hydration, this pathway is determined by the kinetic hindrance of the 0–1 and 1/3–1 transitions during hydration. The hypothetical root cause for the kinetic hindrance of the 1–1/3 transitions is in the dramatic structural change that the crystalline lattice has to undergo during this transition and the resulting high activation energy (Figure 5).

#### The Pathway during Dehydration

Now that the hydration pathway is explained, we focus on the dehydration steps. As mentioned above, there are two regimes. At lower water vapor pressures, the observed pathway is 2–1–0. The first step of the 2–1 dehydration is as expected because they are adjacent hydrates. However, the second step of 1–0 dehydration is not as expected because the tritohydrate is bypassed.

In the phase diagram in Figure 7, it is shown that 1–0 dehydration occurs at higher temperatures than the 0–1 equilibrium line. The 1/3–0 dehydration step occurs at much higher temperatures (∼160–180 °C) than all other phase transition onsets. This could indicate that the 1–0 dehydration step is the favorable step and that the tritohydrate is a metastable compound, as predicted by the DFT calculations (Supporting Information).

With eq 4 and using the fact that $\Delta G_{0\to 1}=\Delta G_{0\to 1/3}+\Delta G_{1/3\to 1}$ , one can show that (eq 6)

$$p_{eq}^{0\to 1}={\left(p_{eq}^{0\to 1/3}\right)}^{1/3}{\left(p_{eq}^{1/3\to 1}\right)}^{2/3}$$

which implies that $p_{eq}^{0\to 1/3}\leq p_{eq}^{0\to 1}\leq p_{eq}^{1/3\to 1}$ .

This explains why the 1–0 transition occurs instead of a 1–1/3 transition.

Above 100 mbar water vapor pressure, both intermediate hydrates (tritohydrate and monohydrate) were detected via the derivative of the sample weight over time (Figure 3). However, the monohydrate is not observed to form a stable plateau like the dihydrate and tritohydrate before the sample dehydrates further to the tritohydrate. The phase diagram from Sinke et al.^22^ suggests that the monohydrate is stable above 180 °C. This indicates that the monohydrate is indeed a stable phase. This indicates that the 1–1/3 transition is no longer kinetically hindered and outcompetes the 1–0 transition.

Finally, to answer the question (IV) about the pathway of the CaCl_2_ dehydration, the tritohydrate cannot be accessed at low water vapor pressures as the 1–0 transition is the preferred pathway from a thermodynamic point of view.

## Conclusion

In this study, the path-dependent hydration–dehydration behavior of CaCl_2_ (0-1/3-2-1-0) was investigated. The main question was why CaCl_2_ has different intermediates between hydration (tritohydrate) and dehydration (monohydrate) below a 100 mbar water vapor pressure and why does this change above this vapor pressure (0-1/3-2-1/3-0)? Therefore, isobaric TGA measurements were done at many different water vapor pressures, and the found (de)­hydration onsets were compared to each other and the known equilibrium lines.

Our experiments show that the difference between hydration and dehydration pathways is a consequence of the poor kinetics of the 1/3-1 transition in both directions. During hydration, it prevents the formation of a monohydrate phase and leads to direct 1/3-2 transitions. During dehydration, it often prevents the formation of tritohydrate and leads to direct 1-0 transitions.

The kinetic hindrance of the 1–1/3 transition can be understood based on the crystal structures of these hydrates. We determined the tri- and monohydrate structures and showed that the 1/3–1 transition implies a complex rearrangement of the crystalline lattice. This demonstrated that, due to the proximity of the trihydrate and monohydrate in terms of temperature and water vapor pressure in the phase diagram, the kinetic hindrance of hydration and dehydration reactions in salt hydrates, such as CaCl_2_ or others described in ref 30 can alter the observable steps during measurements.

## Supplementary Material



## Notes

Additional measurements and calculations including extra TGA data on the CaCl_2_ phase transition onsets, equilibrium vapor pressures of CaCl_2_ hydration steps, additional PXRD measurements to confirm the monohydrate phase during hydration, and details of the SCXRD data of CaCl_2_ hydrates and DFT calculations for thermodynamic predictions (PDF)
