# Supplementary material for: Path-Dependent Hydration and Dehydration of CaCl2
Source: Cryst Growth Des. 2025 Jul 11;25(15):5679–88. doi: 10.1021/acs.cgd.4c00628 (PMC12333002; doi:10.1021/acs.cgd.4c00628)
Supplement: Supplementary file 1 [file cg4c00628_si_001.pdf]

Supporting Information for “Path-dependent hydration and dehydration of $CaCl_2$”

*Michaela C. Eberbach[1,2], Hyerin Seo[1], Aleksandr I. Shkatulov[1,3], Paul Tinnemans[4], Hendrik P. Huinink[*1,2], Hartmut R. Fischer[5] and Olaf C. G. Adan[1,5]*

1 Eindhoven University of Technology, Den Dolech 2, 5600 MB Eindhoven, The Netherlands

2 EIRES, Horsten 1, 5612 AX Eindhoven, The Netherlands

3 Iberian Center for Research in Energy Storage, CIIAE, Polígono 13, Parcela 31, "El Cuartillo", 10004 Cáceres, Spain

4 Radboud University, Houtlaan 4, 6525 XZ Nijmegen, The Netherlands

5 TNO Materials Solutions, High Tech Campus 25, 5656 AE Eindhoven, The Netherlands

KEYWORDS Salt hydrate, phase transitions, crystal structure, hydrate, calcium chloride, tritohydrate, monohydrate, dihydrate and anhydrate.

Description

The measurements and representation of the different $CaCl_2$ phase transition onsets:

Isobaric TGA measurements at different water vapor pressures of $CaCl_2$ powder.

Equilibrium lines of $CaCl_2$ an-, mono-, trito and dihydrates

Additional isobaric semi-isothermal in situ PXRD measurements to confirm the monohydrate during hydration

Additional details of the single crystal data of the $CaCl_2$ hydrates:

The number of ligands and number of water molecule ligands around the calcium ions of the trito- and monohydrate

The space groups, symmetries and dimensions of all known $CaCl_2$ hydrate crystal structures

Ab initio thermodynamic calculations

$CaCl_2$ onsets

Isobaric TGA measurements at different water vapor pressures

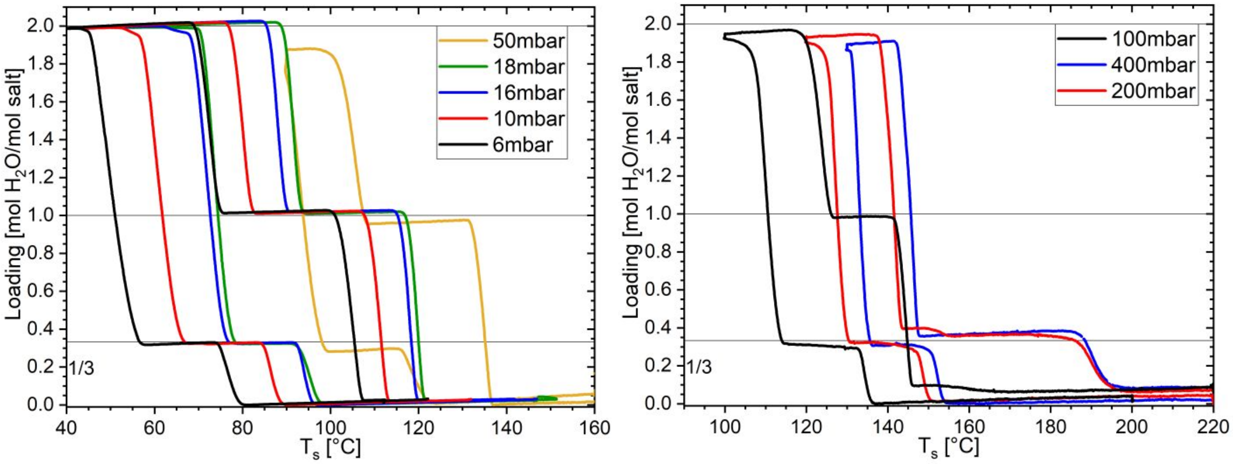

Figure S1 – *pure $CaCl_2$ hydration and dehydration during isobaric measurements at a) 6-50 mbar and b) 100-400 mbar water vapor pressures and 0.2 K/min temperature ramp.*

Equilibrium lines of $CaCl_2$ an-, mono-, trito and dihydrates

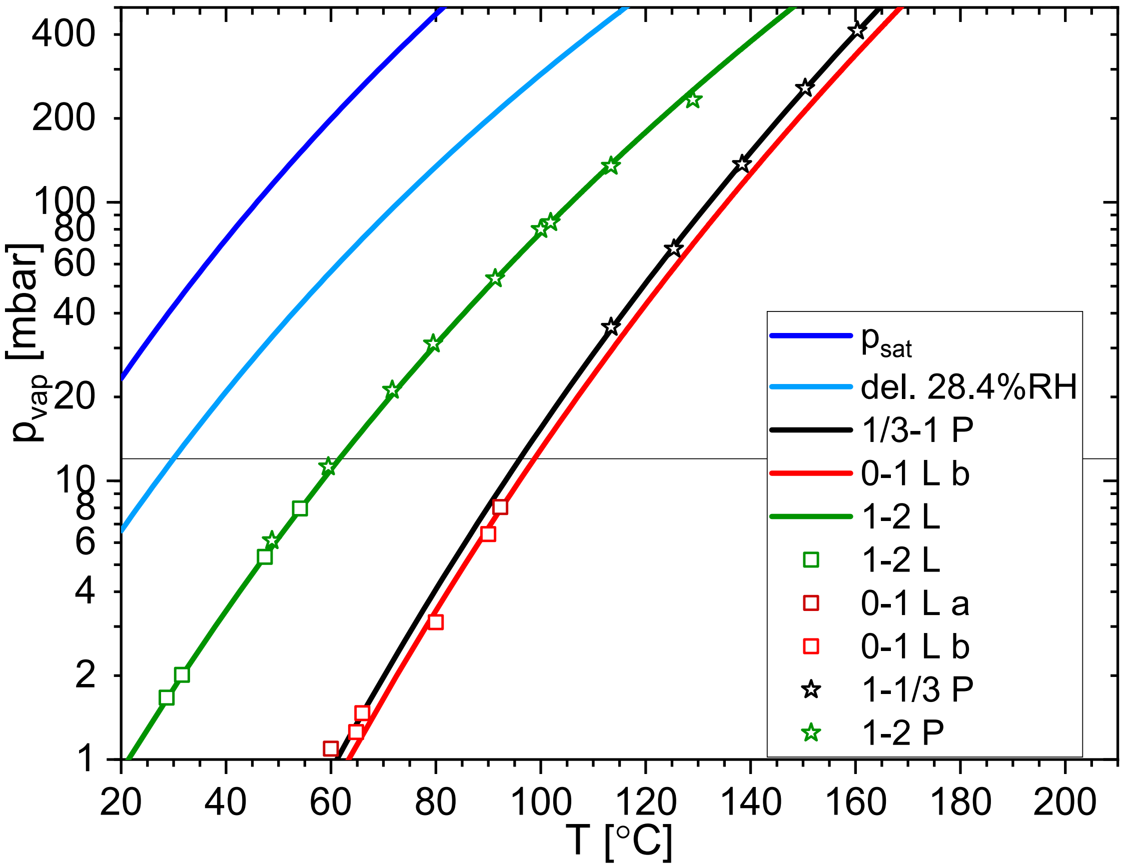

Figure S2 - *The phase diagrams of $CaCl_2$ with water vapor pressure pvap as a function of the temperature T. The data determined by Lannung[1] is labeled with "L" and is shown as solid lines for the equilibrium lines and their data points as empty squares of the same color. The data points and fitted line for the 1/3 -1 transition from Pitzer et al.[2] are labeled with "P" and are depicted as the empty stars and the solid orange/yellow line, respectively. Results obtained with the pT-meter in this work are shown as solid triangles and labeled with "pT".*

Additional PXRD measurements

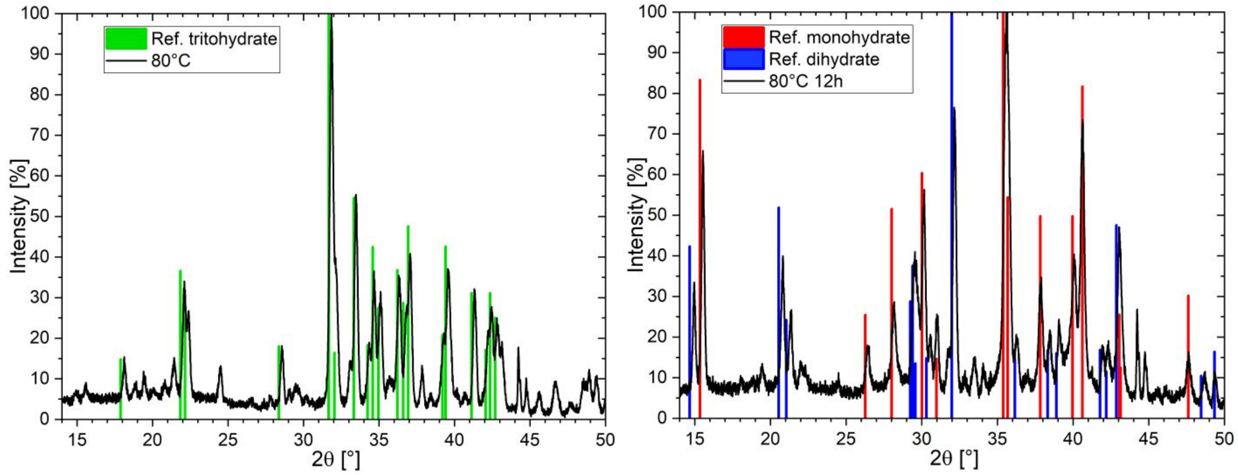

Figure S3 – *Isobaric XRD measurement at 12 mbar water vapor pressure with three pre-cycles from 150-45°C at 1°C/min and subsequent cooling to 80°C after a) 0 and b) 12 hours.*

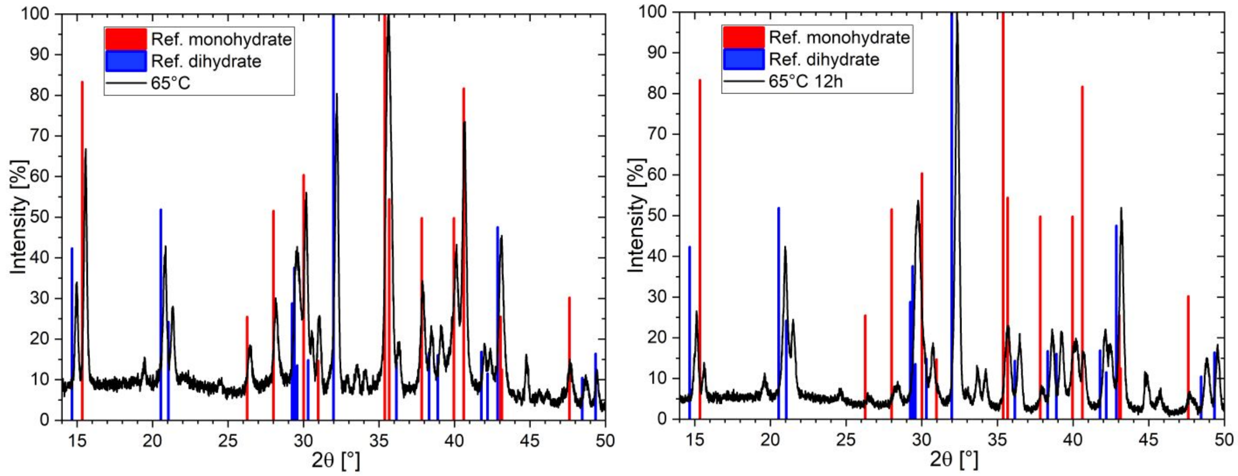

Figure S4 – *Isobaric XRD measurement at 12 mbar water vapor pressure with three pre-cycles from 150-45°C at 1°C/min, subsequent cooling via a 12 hour step at 80°C to 65°C after a) 0 and b) 12 hours.*

Single crystal data

Table S1 - Coordination around Ca ion in different hydrate states.

hydrate	# ligands on Ca ion	# H_2O ligands
anhydrate	6	0
tritohydrate	6 or 7	0 or 0.5
monohydrate	8	2x0.5
dihydrate	6	2
alpha-tetrahydrate	7	4
beta-hydrate	6 or 7	0 or 6
gamma-hydrate	6	4
hexahydrate	9	3 + 6x0.5

Table S2 – The unit cells of the $CaCl_2$ hydrates

hydrate	Space group	symmetry	V [nm^3]	a [Å]	b [Å]	c [Å]
0	Pnnm	Orthorhombic	0.169	6.24	6.43	4.20
1/3	Pnma	Orthorhombic	0.943	10.85	9.91	8.77
1	Pmmn	Orthorhombic	0.185	8.39	3.82	5.77
2	Pbcn	Orthorhombic	0.531	5.89	7.477	12.07
4α	P-1	Triclinic	0.331	6.59	6.37	8.56
4β	$P2_1/c$	Monoclinic	1.060	8.92	10.22	12.79
4γ	$P2_1/c$	Monoclinic	0.391	6.14	7.67	8.90
6	P321	Trigonal	0.212	7.88	7.88	3.95

Ab initio thermodynamic calculations

The ab initio calculations of thermodynamics were carried out by using Density Functional Theory (DFT) and lattice phonon dynamics study.

All density functional theory (DFT) calculations were performed using the Vienna Ab initio Simulation Package (VASP). The Strongly Constrained and Appropriately Normed (SCAN) meta-GGA functional was employed to describe electronic exchange–correlation effects to capture the effects intermediate-range effects of ionic interactions, water molecules coordination and hydrogen bonding.

Core and valence electrons were described by the projector-augmented wave (PAW) pseudopotentials and a plane-wave basis set with an energy cutoff of 500 eV was employed. Brillouin zone integrations were carried out using a Γ-centered k-point grid with resolution 0.04 $Å^{-1}$ in units of 2π/Å ensuring that the total energies were converged to within 1×10^{-9}eV with respect to k-point sampling.

The structures were optimized by using a conjugate-gradient or quasi-Newton algorithm until the residual forces on each atom were smaller than 0.01 eV $Å^{-1}$. During geometry relaxations, both cell parameters and atomic positions were allowed to vary, obtaining a fully relaxed structure at T = 0 K.

Lattice dynamical properties and thermodynamic parameters were computed using the finite displacement (frozen phonon) method as implemented in the Phonopy package. The force

constants were obtained by displacing atoms (by 0.01 Å) symmetrically in a supercell containing a number of primitive unit cells and calculating the atomic forces using the same DFT parameters as described above (Table 1). The phonon dispersion relations were obtained by interpolating the force constant matrix using the Fourier interpolation scheme.

Table 1. Parameters for the thermodynamic properties calculations.

Compound	Unit cell dimensions (XxYxZ)	Number of displacements
$CaCl_2$	2x2x3	6
$CaCl_2 \cdot 1/3H_2O$	1x2x2	42
$CaCl_2 \cdot H_2O$	2x4x2	12
$CaCl_2 \cdot 2H_2O$	2x2x1	27

The vibrational free energy, entropy, and heat capacity were computed within the quasiharmonic approximation (QHA) by considering the temperature dependence of the phonon frequencies. The quasiharmonic calculations were performed by optimizing the crystal structure followed by calculations for force constants and dynamical matrices. Phonopy package was used to derive the thermodynamic parameters. No volume-dependent corrections were used.

The thermodynamic parameters of the transitions were calculated for dehydration reactions:

$$A_{(s)} = B_{(s)} + nH_2O_{(g)}$$

where A and B are crystalline phases. The specific free Gibbs energy was calculated as:

$$\Delta_r G(T) = \Delta_r G^o(T) + nRTln\frac{P_{H_2O}}{P^o}$$

where P^o is standard pressure (1bar). The $\Delta_r G^o$ in turn were calculated as follows:

$$\Delta_r G^o(T) = \Delta E_{DFT} + \Delta E_{ZP} + \Delta F^{PH}(T) - nG^o{}_{H_2O}(T)$$

The ΔE_{DFT} is the energy difference at T = 0K and it was directly derived from DFT calculations for solid phases $CaCl_2$, $CaCl_2{\cdot}1/3H_2O$, $CaCl_2{\cdot}H_2O$, $CaCl_2{\cdot}2H_2O$. The PV contribution to enthalpy was ignored as negligible. The zero-point energy term ΔE_{ZP} and free phonon energies were derived from the phonon frequencies calculations for solid phases, and $G_{H2O}(T)$ term was calculated for water molecules by using DFT calculation for a H_2O molecule in a 30Åx30Åx30Å box (T = 0 K) further followed by calculating the temperature dependency of enthalpy by using the ideal gas approximation, the literature values for entropy were used to calculate free Gibbs energy.

Calculated equilibria

The equilibrium lines $\Delta_r G(T)$ were calculated ab initio for all six combinations of phases, namely, for 2-0, 1-0, 1/3-0, 2-1, 1-1/3 and 2-1/3 transitions (Fig. S4) at $P(H_2O)$ = 12 mbar and 400 mbar.

Figure S4 (left) shows the sequence of phase transitions at $P(H_2O)$ = 12 mbar. If we start with $CaCl_2{\cdot}2H_2O$ at 273K and rise the temperature going along the $\Delta_r G = 0$ horizontal line, we will cross the green line (the beads on the graph) and 2-1 transition will occur. Then our system will consist of $CaCl_2{\cdot}H_2O + H_2O$. Upon further increase of temperature, we will cross magenta, grey and blue lines (2-1/3, 2-0 and 1/3-0 transition, respectively), but since we do not have neither $CaCl_2{\cdot}2H_2O$ nor $CaCl_2{\cdot}1/3H_2O$ in our system, no process will take place. Then the red line of 1-0 transition is crossed and the system turns into ($CaCl_2 + H_2O$). Finally, crossing the purple line 1-1/3 will not have any effect. **Thus,** DFT predicts that the sequence of equilibria states at $P(H_2O)$

= 12 mbar is 2-1-0. If we start from anhydrate, then DFT predicts that the equilibrium sequence is 0-1-2.

The fact that we experimentally observe 0-1/3-2 sequence during hydration shows that the thermodynamically favored 0-1 and 1/3-1 transitions are hindered kinetically. Indeed, if we consider the next-left line to the red that contains anhydrate as one of the involved compounds, we will encounter the blue line (1/3-0) which will correspond to 0-1/3 transition, which should immediately be followed by 1/3-1 transition since we are to the left of the purple 1-1/3 line. Thus, both 0-1 and 1/3-1 transitions are likely to be hindered kinetically. Finally, if still having $CaCl_2 \cdot 1/3H_2O + H_2O$ at the intersection of blue line and $\Delta_r G = 0$ (due to the kinetic hindrances) we move to the left, the magenta line 2-1/3 will be crossed, and this transition is observed experimentally.

Figure S4 (right) shows the sequence of phase transitions at $P(H_2O)$ = 400 mbar. After similar consideration one can conclude that DFT predicts that 2-1-0 is the sequence of equilibrated phases. After crossing the green 2-1 line the dihydrate should turn into monohydrate, however, this transition does not always happen, likely, due to kinetic hindrance of 2-1 transition. If we keep the dihydrate phase and increase the temperature (i.e. move right to the green line) we will cross the yellow line of 2-1/3 transition that is observed experimentally. Then crossing the blue line 1/3-0 at higher T completes the picture and finalizes the 2-1/3-0 sequence.

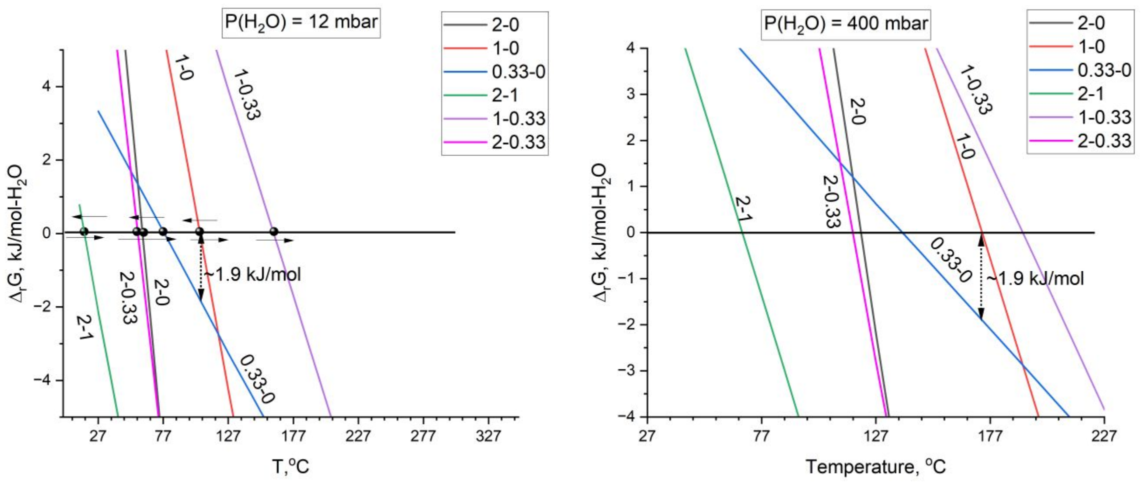

Figure S5 - The change of Gibbs energy $\Delta_r G$ for all six transitions between hydrates.

References

[1] A. Lannung, 1936, Dampfdruckmessungen des Systems Calciumchlorid-Wasser, Zeitschrift für anorganische und allgemeine Chemie, Vol. 228, pp. 1-18, DOI: https://doi.org/10.1002/zaac.19362280102.

[2] K. S. Pitzer and C. S. Oakes, 1994, Thermodynamics of calcium chloride in concentrated aqueous solutions and crystals, Journal of Chemical and Engineering Data, Vol. 39, pp. 553-559, DOI: https://doi.org/10.1007/BF00650677.
